# Inhibition of the SK-N-MC human neuroblastoma cell line *in vivo* and *in vitro* by a novel nutrient mixture

**DOI:** 10.3892/or.2013.2307

**Published:** 2013-02-27

**Authors:** M. WAHEED ROOMI, TATIANA KALINOVSKY, NUSRATH W. ROOMI, ALEKSANDRA NIEDZWIECKI, MATTHIAS RATH

**Affiliations:** Dr Rath Research Institute, Oncology Division, Santa Clara, CA, USA

**Keywords:** neuroblastoma, SK-N-MC, tumor growth, MMP-2, MMP-9, apoptosis

## Abstract

Neuroblastoma, a peripheral nervous system cancer that can be highly invasive and metastatic, accounts for 8–10% of all solid childhood tumors in children under the age of 15 years. Despite multiple clinical efforts, prognosis remains poor for this enigmatic disease. A nutrient mixture (NM) containing lysine, proline, ascorbic acid and green tea extract has shown significant antitumor effects. Using the nude mouse xenograft model, we investigated the efficacy of NM. We also tested the effect of NM *in vitro*, evaluating cell viability, secretion of the matrix metalloproteinases (MMP)-2 and MMP-9, tissue inhibitor of metalloproteinase (TIMP)-2 secretion, Matrigel invasion and cellular apoptosis and morphology. Athymic nude mice 5–6 weeks of age were inoculated with 3×10^6^ SK-N-MC neuroblastoma cells subcutaneously and randomly divided into two groups. Group A was fed a regular diet and group B a regular diet supplemented with 0.5% NM. Four weeks later, the mice were sacrificed and their tumors were excised, weighed and processed for histology. We also tested the effect of NM *in vitro*. NM inhibited the growth of xenograft tumors by 22% (P=0.04); and, *in vitro*, NM induced dose-dependent inhibition of cell proliferation with a decrease of 27% (P=0.001) and 36% (P=0.002) at 500 and 1000 μg/ml NM compared to the control, respectively. Zymography revealed MMP-2 secretion in normal cells and PMA (100 ng/ml)-induced MMP-9 secretion. NM inhibited the secretion of both MMPs with total blockage at a concentration of 100 μg/ml. Reverse zymography demonstrated a dose-dependent increase in TIMP-2 expression by NM. Notable, SK-N-MC human neuroblastoma cells were not invasive through Matrigel. NM induced dose-dependent apoptosis of SK-N-MC cells. The results suggest that NM may have therapeutic potential in treating neuroblastoma.

## Introduction

Neuroblastoma, the most common solid extracranial tumor in children, accounts for 7% of pediatric cancers (1). Approximately 650 new cases of neuroblastoma are diagnosed each year in the United States. The cancer is usually diagnosed at 1 to 2 years of age and 90% of cases are diagnosed by 5 years of age (1). This embryonal cancer typically arises from the adrenal medulla or paraspinal sympathetic ganglia of the abdomen, chest or neck and often metastasizes to the liver, regional lymph nodes, bone marrow and bone (2). Neuroblastoma tumors that are benign, localized and well differentiated are successfully treated by surgical resection. Still, a majority of neuroblastoma patients develop an aggressive disease that is refractory to intensive therapies. Current treatment for high risk neuroblastoma has reached an extreme toxic and virtually intolerable level that includes intensive chemotherapy, radiotherapy, autologous bone marrow transplantation and retinoid and immunomodulation among others (3). Despite aggressive conventional treatments, the majority of children older than one year of age with advanced stage neuroblastoma die from progressive disease, and only 40% of children over 4 years of age survive for 5 years, emphasizing an urgent need for the development of innovative effective treatment strategies (4).

Advanced stages of neuroblastoma show increased expression of the matrix metalloproteinase (MMP-2), and a higher MMP-2 to TIMP-2 ratio has been shown to correlate with poorer prognosis for neuroblastoma patients (5). Sugiura *et al*(6) reported higher levels of MMP-2 and MMP-9 in patients with stage IV (metastatic) disease when compared with those in stages I and II (non-invasive and non-metastatic). MMP-2 was present in both tumor and stromal cells; however, MMP-9 was present in stromal, vascular and perivascular cells surrounding nests of tumor cells.

We have developed strategies to inhibit cancer development and its spread using naturally occurring nutrients such as lysine, proline, ascorbic acid and green tea extract [nutrient mixture (NM)]. This nutrient mixture has exhibited synergistic anticancer activity *in vivo* and *in vitro* in a number of cancer cell lines through inhibition of cancer cell growth, MMP secretion, invasion, metastasis and angiogenesis (7–9). Our main objective in this study was to evaluate the effectiveness of NM on neuroblastoma cells *in vivo* using the nude mouse xenograft model and *in vitro*, evaluating the effect of NM on cell viability, MMP-2 and -9 secretion, TIMP-2 secretion, Matrigel invasion and cellular apoptosis and morphology.

## Materials and methods

### In vivo

#### Animals

Male athymic mice (NCr-nu/nu), ~5 weeks of age on arrival, were purchased from Simonsen Laboratories, Gilroy, CA, USA and maintained in microisolator cages under pathogen-free conditions on a 12-h light/12-h dark schedule for one week. All procedures were performed according to humane and customary care and use of experimental animals and followed a protocol approved by the internal institutional animal safety review committee.

#### Experimental design

After housing for a week, the mice (n=16) were inoculated subcutaneously with 3×10^6^ neuroblastoma SK-N-MC cells in 0.2 ml PBS and 0.1 ml Matrigel (BD Bioscience, Bedford, MA, USA). After injection, the mice were randomly divided into two groups of 8 mice each; group A mice were fed regular Purina mouse chow and group B the regular diet supplemented with 0.5% NM (w/w). The regular diet was Laboratory Rodent Diet 5001 from Purina Mills, Inc. LLC/TestDiet^®^ (Gray Summit, MO, USA). The 0.5% NM diet was milled and pressed by Purina Mills and generated by Vita-Tech (Tustin, CA, USA). During the study, the mice consumed, on the average, 4 g of their respective diets/day. Thus, the supplemented mice received ~20 mg of NM/day. After four weeks, the mice were sacrificed and their tumors were excised, weighed and processed for histology. The mean weight of mice at initiation of the study and termination of the study did not differ significantly between the groups.

#### Histology

Tissue samples were fixed in 10% buffered formalin. All tissues were embedded in paraffin and cut at 4–5 μm. Sections were deparaffinized through xylene and graduated alcohol series to water and stained with hematoxylin and eosin (H&E) for evaluation using a standard light microscope.

### In vitro studies

#### Cell culture

Human neuronal epithelioma SK-N-MC cells (ATCC) were grown in MEM, supplemented with 10% fetal bovine serum, penicillin (100 U/ml) and streptomycin (100 mg/ml) in 24-well tissue culture plates (Costar, Cambridge, MA, USA). Cells were incubated with 1 ml of media at 37°C in a tissue culture incubator equilibrated with 95% air and 5% CO_2_. At near confluence, the cells were treated with the nutrient mixture, dissolved in media and tested at 0, 10, 50, 100, 500 and 1,000 μg/ml in triplicate at each dose. Phorbol 12-myristate 13-acetate (PMA) (100 ng/ml) was added to the cells to induce MMP-9 secretion. The plates were then returned to the incubator.

#### MTT assay

Cell viability was evaluated by MTT assay, a colorimetric assay based on the ability of viable cells to reduce a soluble yellow tetrazolium salt [3-(4,5-dimethylthiazol-2-yl) 2,5-diphenyl tetrazolium bromide] (MTT) to a blue formazan crystal by mitochondrial succinate dehydrogenase activity of viable cells. This test is a good index of mitochondrial activity and thus of cell viability. After a 24-h incubation, the cells were washed with phosphate-buffered saline (PBS) and 500 μl of MTT (#M-2128; Sigma) 0.5 mg/ml in media was added to each well. After MTT addition (0.5 mg/ml), the plates were covered and returned to the 37°C incubator for 2 h, the optimal time for formazan product formation. Following incubation, the supernatant was carefully removed from the wells, the formazan product was dissolved in 1 ml DMSO, and absorbance was measured at 570 nm in the BioSpec 1601 Shimadzu spectrometer. The OD_570_ of the DMSO solution in each well was considered to be proportional to the number of cells. The OD_570_ of the control (treatment without supplement) was considered 100%.

#### Gelatinase zymography

Gelatinase zymography was performed in 10% Novex Pre-Cast SDS polyacrylamide gel (Invitrogen) in the presence of 0.1% gelatin under non-reducing conditions. Culture media (20 μl) were mixed with sample buffer and loaded for SDS-PAGE with Tris glycine SDS buffer as suggested by the manufacturer (Novex). Samples were not boiled before electrophoresis. Following electrophoresis the gels were washed twice in 2.5% Triton X-100 for 30 min at room temperature to remove SDS. The gels were then incubated at 37°C overnight in substrate buffer containing 50 mM Tris-HCl and 10 mM CaCl_2_ at pH 8.0 and stained with 0.5% Coomassie Blue R-250 in 50% methanol and 10% glacial acetic acid for 30 min and destained. Upon renaturation of the enzyme, the gelatinases digest the gelatin in the gel and provide clear bands against an intensely stained background. Protein standards were run concurrently, and approximate molecular weights were determined by plotting the relative mobilities of known proteins.

#### Reverse zymography

TIMPs were analyzed by reverse zymography on 15% SDS gels containing serum-free conditioned medium from cells. After electrophoresis the gels were washed twice with 2.5% Triton X-100 for 30 min at room temperature to remove SDS. The gels were then incubated at 37°C overnight in 50 mM Tris-HCl and 10 mM CaCl_2_ at pH 7.6 and stained with 0.5% Coomassie Blue R-25, destained and scanned.

#### Scanning of gelatinase and reverse zymograms

Gelatinase and reverse zymograms were scanned using CanoScan 9950F Canon scanner at 300 dpi. The intensity of the bands was evaluated using the pixel-based densitometer program Un-Scan-It, version 5.1, 32-bit, by Silk Scientific, Inc. (Orem, UT, USA), at a resolution of 1 scanner unit (1/100 of an inch for an image that was scanned at 100 dpi). The pixel densitometer calculates the optical density of each pixel (values 0 to 255) using the darkly stained background of the gel as a pixel value of 0. A logarithmic optical density scale was used since the optical density of films and gels is logarithmically proportional to the concentration. The pixel densitometer sums the optical density of each pixel to give a band’s density. In all graphs, band densities were reported as percentages of the sums of all pixels in a given lane (treatment) of a gel.

#### Matrigel invasion

Invasion studies were conducted using Matrigel (Becton-Dickinson) inserts in 24-well plates. Suspended in medium, SK-N-MC cells were supplemented with nutrients, as specified in the design of the experiment and seeded on the insert in the well. Thus, both the medium on the insert and in the well contained the same supplements. The plates with the inserts were then incubated in a culture incubator equilibrated with 95% air and 5% CO_2_ for 24 h. After incubation, the media from the wells were withdrawn. The cells on the upper surface of the inserts were gently scrubbed away with cotton swabs. The cells that had penetrated the Matrigel membrane and migrated onto the lower surface of the Matrigel were stained with H&E and visually counted under a microscope.

#### Morphology and apoptosis

Morphology of cells cultured for 24 h in test concentrations of NM were evaluated by H&E staining and observed and photographed by microscopy. At near confluence, SK-N-MC cells were challenged with NM dissolved in media at 0, 50, 100, 250, 500 and 1,000 μg/ml and incubated for 24 h. The cell culture was washed with PBS and treated with the caspase reagent as specified in the manufacturer’s protocol (Molecular Probes Image-IT™ Live Green Poly Caspases Detection Kit 135104; Invitrogen). The cells were photographed under a fluorescence microscope and counted. Green-colored cells represented viable cells, while yellow-orange colored cells were early apoptotic and red, late apoptotic

#### Statistical analysis

Data are expressed as means ± SD, as indicated in the results, for the groups. Data were analyzed by independent sample t-test. Pearson’s correlation coefficients were determined for toxicity and invasion correlations to NM concentration using MedCalc Software (Markakerke, Belgium).

## Results

### In vivo

#### Tumor growth

**Figure 1:**
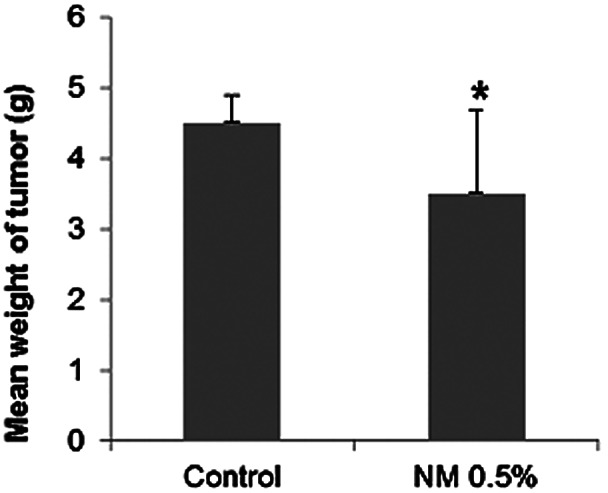
Effect of NM on the growth of human neuroblastoma SK-N-MC subcutaneous xenograft tumors in male nude mice. The comparative mean weights of tumors in the groups are indicated. NM inhibited the mean tumor weight by 22% (P=0.04) with respect to the control group (^*^P<0.05).

**Figure 2:**
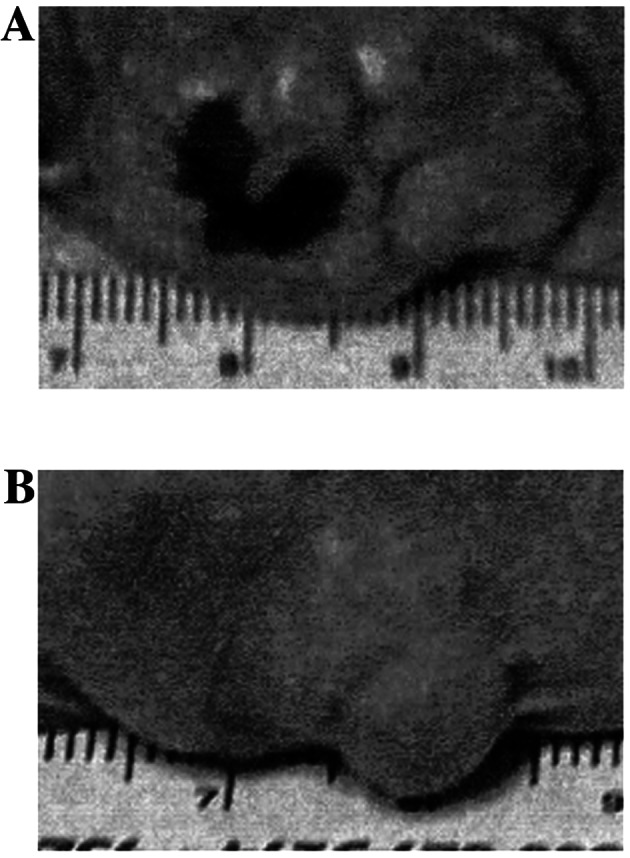
Effect of NM on the growth of human neuroblastoma SK-N-MC subcutaneous xenograft tumors in male nude mice. Representative images of gross tumors from (A) control and (B) 0.5% NM supplemented groups.

NM supplementation significantly inhibited neuroblastoma SK-N-MC xenograft tumor growth. The mean weight of tumors in the nude mice fed the 0.5% NM supplement was inhibited by 22% (P=0.04) in comparison to that of the control group of mice (Figs. 1 and 2).

#### Histopathology

**Figure 3:**
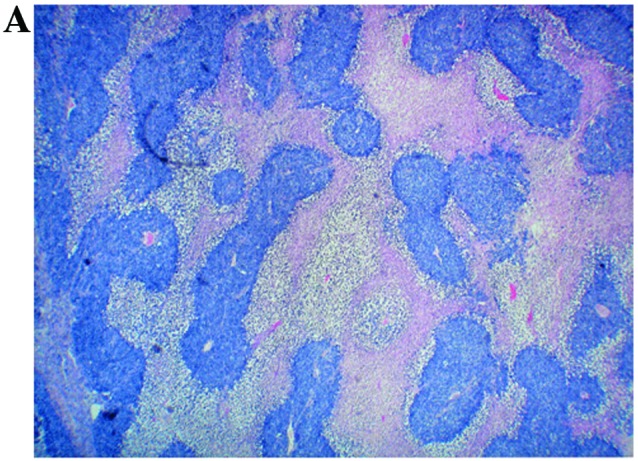

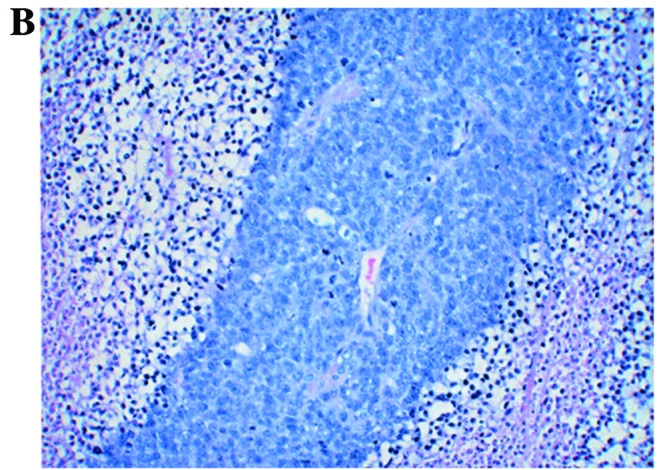

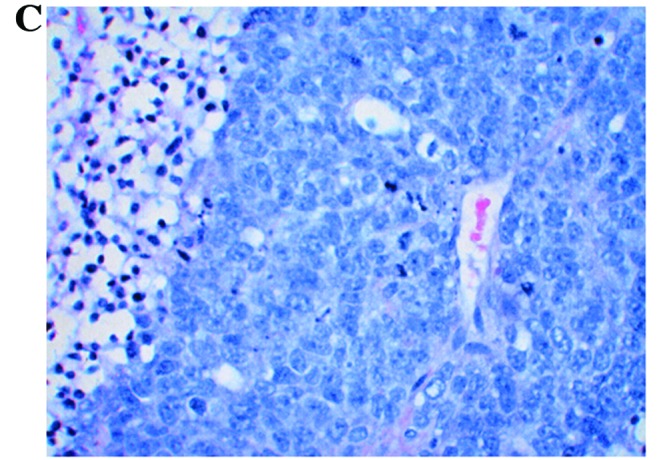

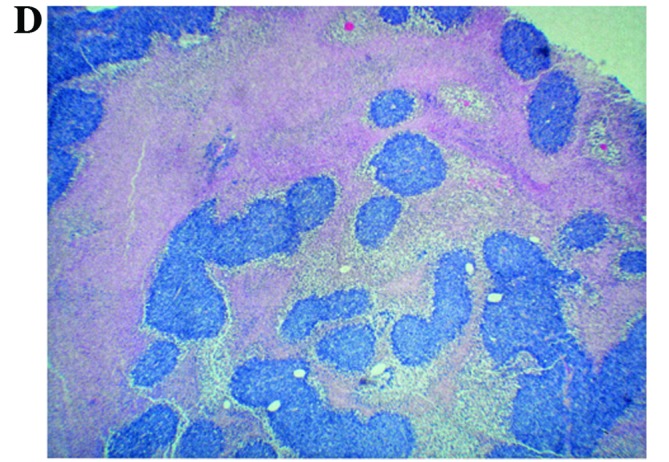

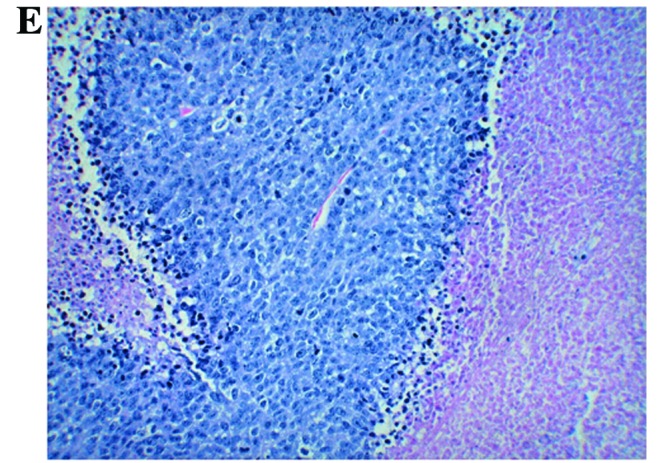

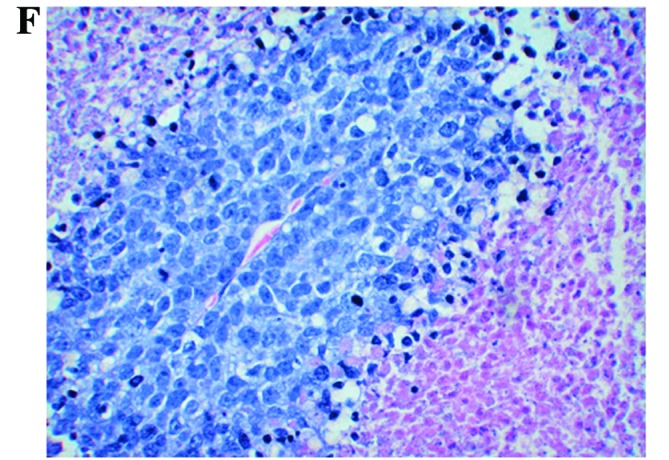
Histopathology of human neuroblastoma SK-N-MC subcutaneous xenograft tumors in the groups. Representative photomicrographs: (A) control, ×40; (B) control, ×200; (C) control, ×400; (D) NM 0.5%, ×40; (E) NM 0.5%, ×200; (F) NM 0.5%, ×400. Histologically, the tumors from both groups were composed of necrotic, expansile, subcutaneous neoplastic masses consistent with neuroblastoma.

Histologically the tumors from both groups were composed of necrotic, expansile, subcutaneous neoplastic masses consistent with neuroblastoma (Fig. 3).

### In vitro

#### Cytotoxicity

**Figure 4:**
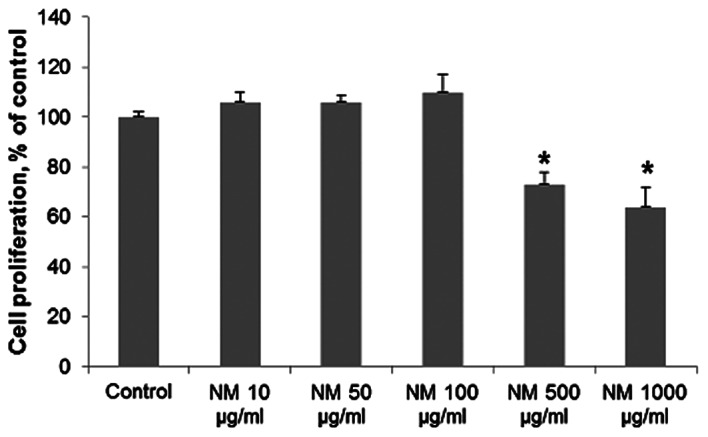
Effect of NM on the viability of neuroblastoma SK-N-MC cells at 24 h as determined by MTT assay. NM exhibited no toxicity to human neuroblastoma SK-N-MC cells at low concentrations of NM, but cytotoxicity of 27% (P=0.001) was evident at 500 μg/ml NM and 36% (P=0.002) at 1,000 μg/ml NM.

NM exhibited no toxicity to human neuroblastoma SK-N-MC cells at low concentrations of NM, but cytotoxicity of 27% (P=0.001) was evident at 500 μg/ml NM and 36% (P=0.002) at 1,000 μg/ml NM (Fig. 4).

#### Gelatinase zymography

**Figure 5:**
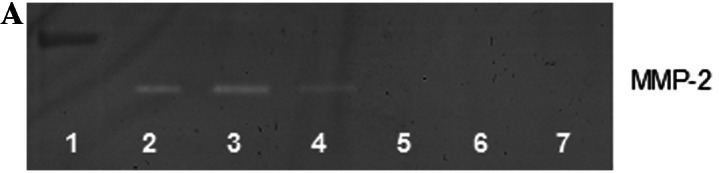

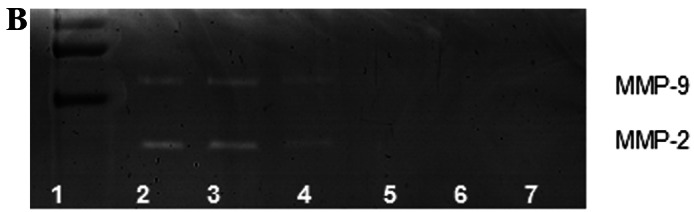

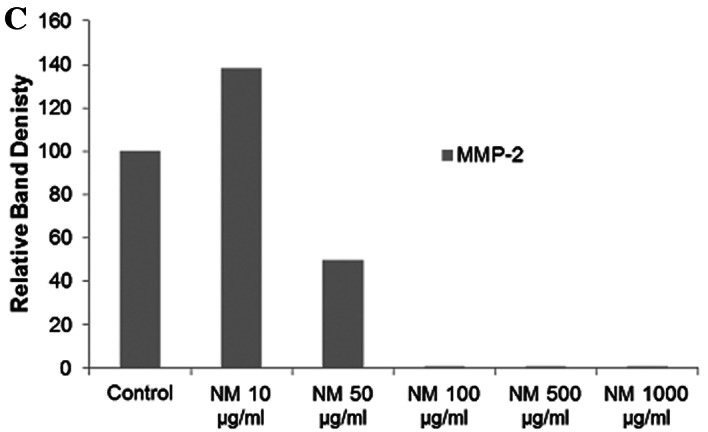

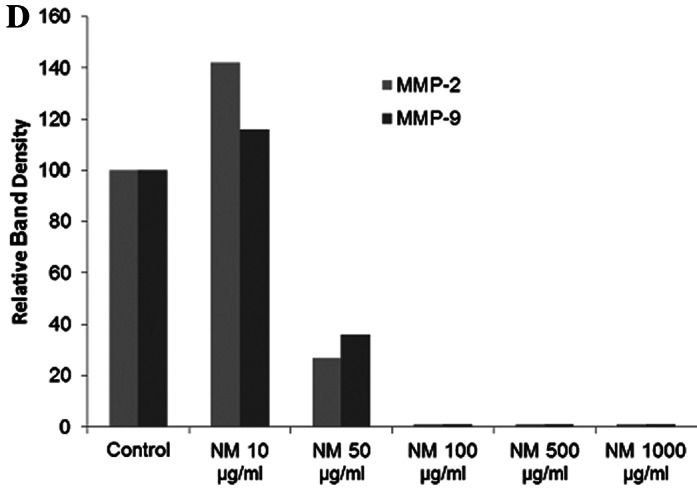
Effect of NM on SK-N-MC cell secretion of MMP-2 and MMP-9 by (A) normal cells and (B) PMA (100 ng/ml)-treated cells. Lane 1, markers; lane 2, control; lanes 3–7, NM 10, 50, 100, 500 and 1,000 μg/ml. Densitometry analysis of (C) uninduced SK-N-MC cells and (D) PMA-treated SK-N-MC cells. Zymography showed a faint band corresponding to MMP-2 secretion, and PMA (200 ng/ml)-induced MMP-9 secretion. NM inhibited the secretion of both MMP-2 and -9 with total blockage at 100 μg/ml concentration.

Zymography showed a faint band corresponding to MMP-2 secretion, and PMA (100 ng/ml)-induced MMP-9 secretion. NM inhibited the secretion of both MMP-2 and -9 with total blockage at a concentration of 100 μg/ml (Fig. 5). MMP-2 secretion by normal SK-N-MC cells was inhibited by 50% by 50 μg/ml NM, and virtually blocked by NM 100–1,000 μg/ml (linear trend R^2^=0.756). Secretion of MMP-2 by PMA-treated cells was inhibited by 73% at 50 μg/ml NM and virtually blocked at 100–1,000 μg/ml NM (linear trend R^2^=0.691). MMP-9 secretion by PMA-treated cells was inhibited by 64% at 50 μg/ml NM and virtually blocked at 100–1,000 μg/ml NM (linear trend R^2^=0.791).

#### TIMP-2

**Figure 6:**
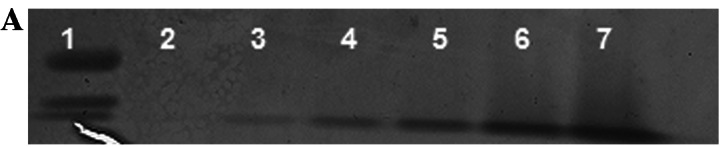

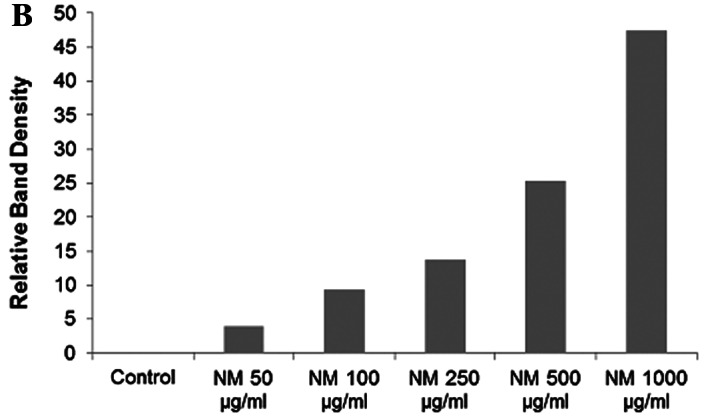
Effect of NM on SK-N-MC cell secretion of TIMP-2. (A) Reverse zymogram: lane 1, markers; lane 2, control; lanes 3–7, NM 10, 50, 100, 500 1,000 μg/ml and (B) densitometry analysis. Reverse zymography revealed upregulation of TIMP-2 activity following NM treatment of SK-N-MC cells in a dose-dependent manner, with minimum activity expressed at 50 and maximum activity at 1,000 μg/ml NM (linear trend R^2^=0.877).

Reverse zymography revealed upregulation of TIMP-2 activity following NM treatment of SK-N-MC cells in a dose-dependent manner, with minimum activity expressed at 50 and maximum activity at 1,000 μg/ml NM (linear trend R^2^=0.877). Reverse zymogram and densitometry analysis are shown in Fig. 6.

#### Correlation of MMP-2 and TIMP-2

**Figure 7:**
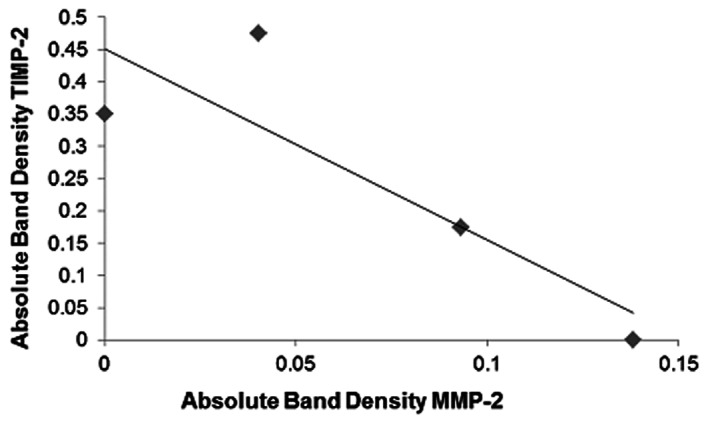
Correlation between secretion of MMP-2 and TIMPs by NM-treated SK-N-MC cells. A negative correlation (correlation coefficient r=−0.8646) was found between MMP-2 and TIMP-2 expression in NM-treated SK-N-MC cells.

A negative correlation (correlation coefficient r=−0.8646) was found between MMP-2 and TIMP-2 expression in the NM-treated SK-N-MC cells (Fig. 7).

#### Matrigel invasion

Notably, human neuroblastoma SK-N-MC cells were not invasive through Matrigel.

#### Cell morphology and apoptosis

**Figure 8:**
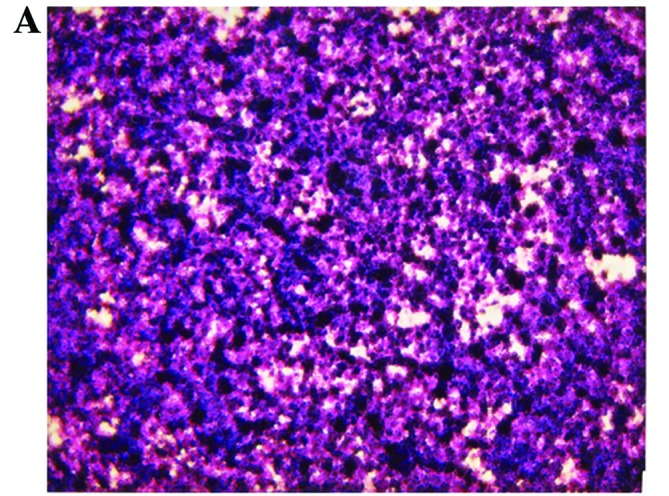

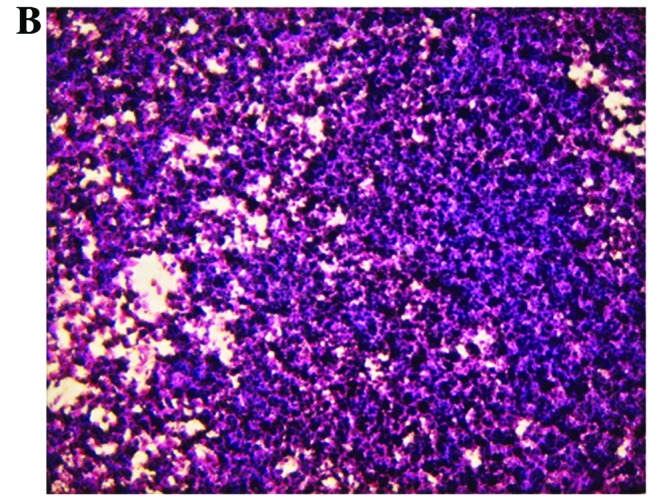

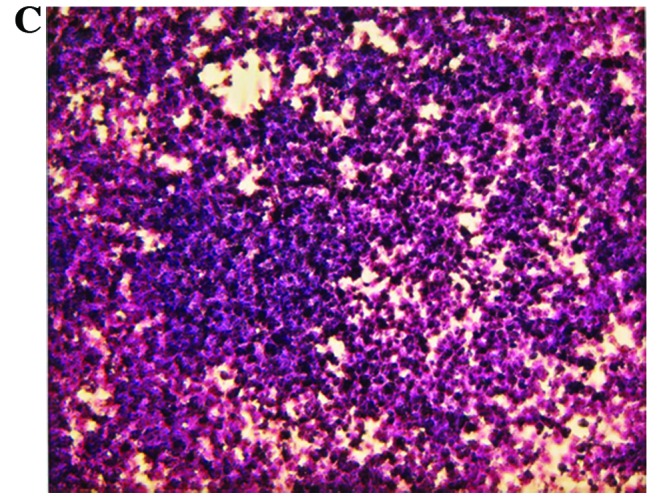

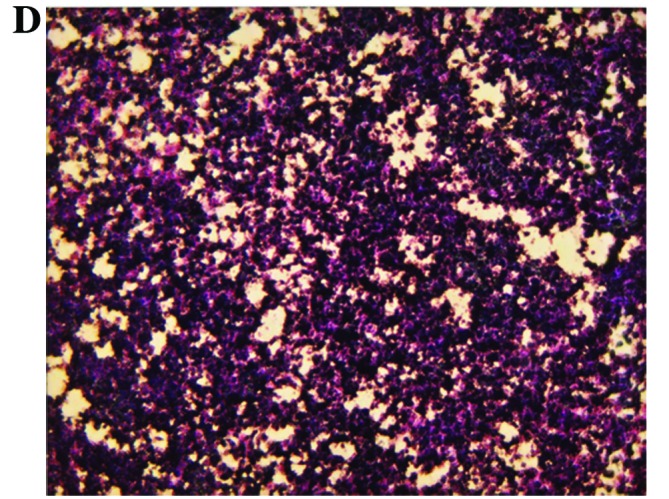

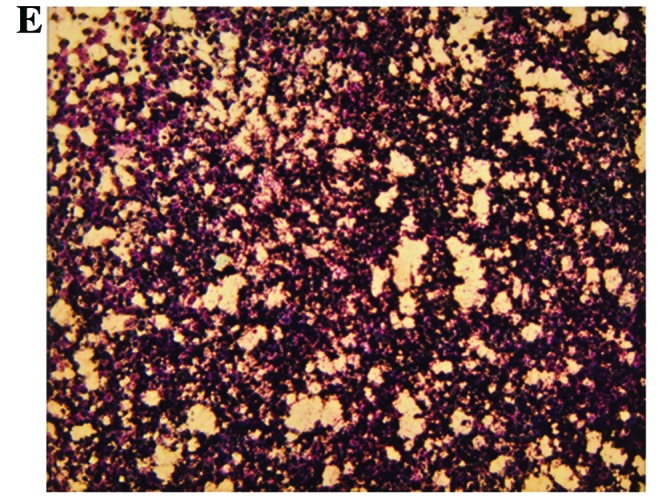
Effect of NM on the morphology of SK-N-MC cells as detected by H&E staining. Neuroblastoma cells exposed to various concentrations of NM indicated no morphological changes at concentrations <500 μg/ml. (A) Control, (B) NM 50 μg/ml, (C) NM 100 μg/ml, (D) NM 500 μg/ml and (E) NM 1,000 μg/ml.

**Figure 9:**
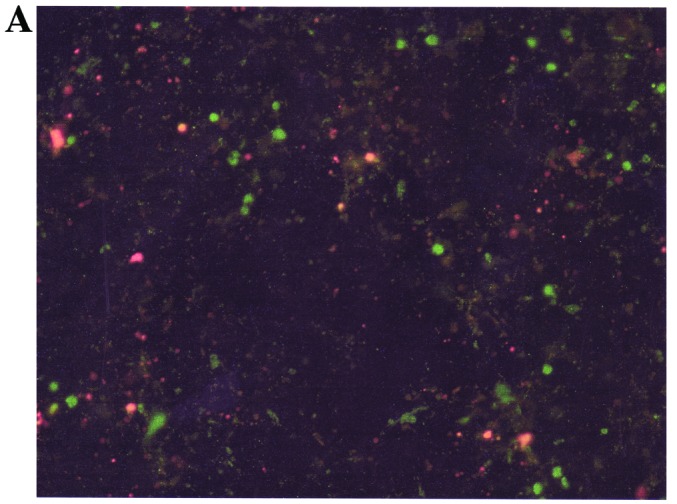

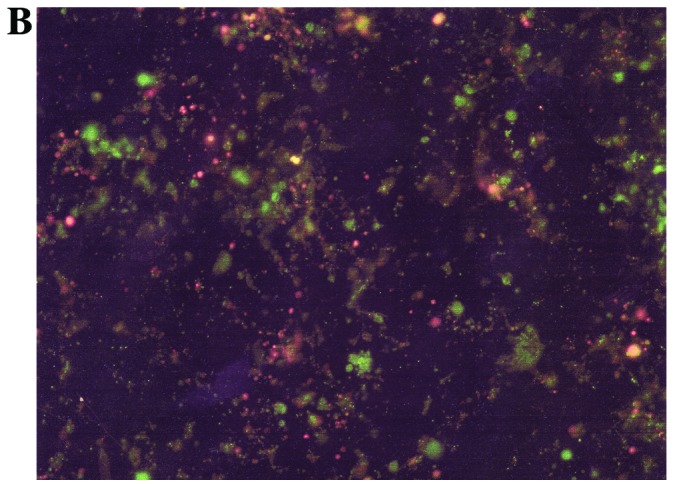

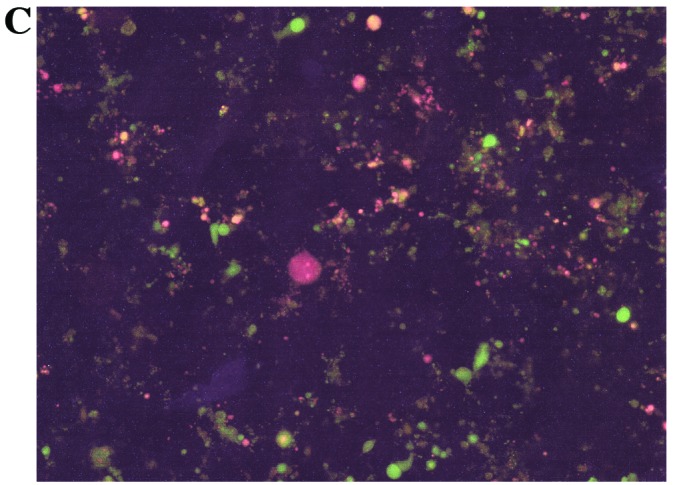

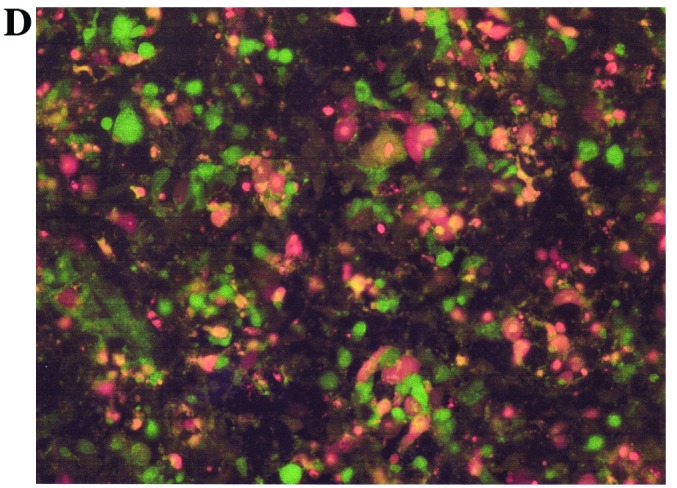

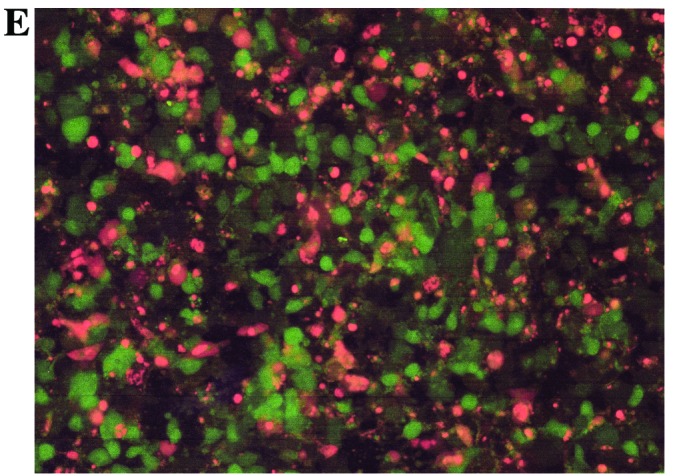

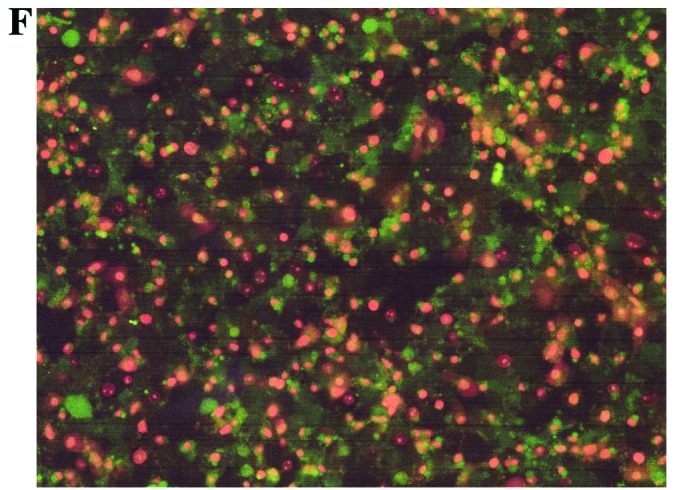
Effect of NM on the apoptosis of SK-N-MC cells. Dose-dependent apoptosis of neuroblastoma cells was evident following NM challenge. (A) Control, (B) NM 50 μg/ml, (C) NM 100 μg/ml, (D) NM 250 μg/ml, (E) NM 500 μg/ml and (F) NM 1000 μg/ml.

**Figure 10:**
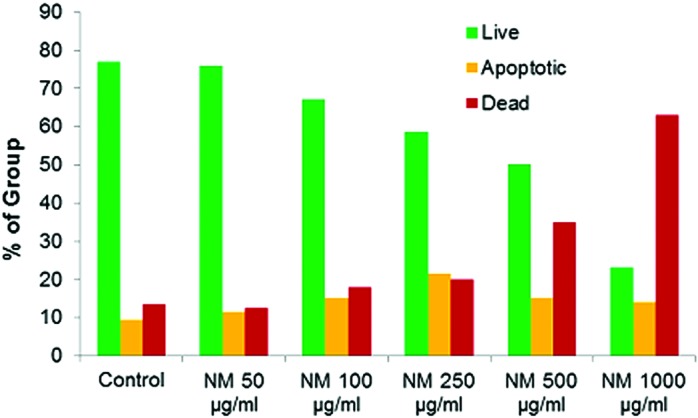
Effect of NM on the apoptosis of SK-N-MC cells; statistical analysis.

Neuroblastoma cells exposed to various concentrations of NM indicated no morphological changes at concentrations <500 μg/ml as detected by H&E staining (Fig. 8). Using the Live Green Poly Caspases Detection kit, dose-dependent apoptosis of neuroblastoma cells was evident following NM challenge (Fig. 9). At 100 μg/ml NM, 67% of cells were viable, 15% of cells were early apoptotic and 18% of cells were late apoptotic. At 500 μg/ml NM, 50% of cells were viable, 15% of cells were early apoptotic, and 35% of cells were late apoptotic. At 1,000 μg/ml NM, 23% of cells were viable, 14% of cells were early apoptotic and 63% of cells were late apoptotic (Fig. 10).

## Discussion

Dietary supplementation with 0.5% NM resulted in a 22% reduction in tumor growth in immune impaired (athymic) male nude mice after subcutaneous administration of 3×10^6^ human neuroblastoma SK-N-MC cells. Results from the cellular proliferation and apoptosis studies support the *in vivo* results, as NM showed dose-dependent toxicity in SK-N-MC cells and induced apoptosis in a dose-dependent manner, with 36% inhibition of cell growth and apoptotic induction of 77% in cells exposed to 1,000 μg/ml NM.

Malignant neuroblastoma is a highly vascularized solid tumor that requires access to blood vessels for growth, invasion and metastasis, and angiogenesis plays an important role in determining tumor phenotype (10). High tumor vascularity is correlated with widely disseminated disease and poor histology and outcome in contrast to low tumor vascularity, which is associated with favorable prognosis, such as localized disease and favorable histology. Thus, researchers are focusing on targeting angiogenesis for the treatment of neuroblastoma (10). Ribatti *et al*(11) reviewed the progress in pre-clinical and clinical research of anti-angiogenic tumor therapy for neuroblastoma. Angiogeneis is mediated by multiple regulating factors, such as growth factors, adhesion molecules and matrix degrading enzymes. In a previous study, NM significantly (P<0.05) reduced bFGF-induced angiogenesis [utilizing a chorioallantoic membrane (CAM) assay] in chick embryos, as well as decreased human osteosarcoma U2OS cell expression of VEGF, angiopoietin-2, bFGF, PDGF and TGFβ-1 (7).

Net matrix degradation and proteolysis depend on the critical local balance between MMPs and TIMP-2. Ara *et al*(5) reported that examination of tumor tissues of 25 neuroblastoma patients for levels of MMPs and TIMP-2 and correlation with stage of disease, revealed poor prognosis with elevated MMP-2 expression and significantly higher advanced stages of neuroblastoma with increased ratios of MMP-2/TIMP-2. In the present study, NM demonstrated dose-dependent inhibition of MMP-2 and -9 secretion by normal and PMA-treated cells with total blockage of both MMPs at 100 μg/ml NM. Furthermore, NM upregulated TIMP-2 activity in SK-N-MC cells in a dose-dependent manner, with minimum activity expressed at 50 and maximum activity at 1,000 μg/ml NM. A negative correlation (correlation coefficient r=−0.8646) was found between MMP-2 and TIMP-2 expression in the NM-treated SK-N-MC cells. The ratio of MMP-2/TIMP-2 expression decreased significantly with increased NM dose: 11.9 at 50 μg/ml NM, 1.9 at 100 μg/ml NM and 0 at 250–1,000 μg/ml NM.

NM was formulated by defining critical physiological targets in cancer progression and metastasis, such as ECM integrity and MMP activity. Adequate supplies of ascorbic acid and the amino acids lysine and proline ensure proper synthesis and hydroxylation of collagen fibers for optimal ECM structure. Manganese and copper are also essential for collagen formation. Lysine, a natural inhibitor of plasmin-induced proteolysis, plays an important role in ECM stability (12,13). Green tea extract has been shown to modulate cancer cell growth, metastasis, angiogenesis, and other aspects of cancer progression (14–18). N-acetyl cysteine has been shown to modulate MMP-9 and invasive activities of tumor cells (19,20). Selenium has been shown to inhibit MMP secretion, tumor invasion, and migration of endothelial cells through ECM (21). Ascorbic acid demonstrates cytotoxic and antimetastatic actions on neuroblastoma and other malignant cell lines (22–27), and cancer patients have been found to have low levels of ascorbic acid (28,29). Low levels of arginine, a precursor of nitric oxide (NO), can limit the production of NO, which has been shown to predominantly act as an inducer of apoptosis (30).

In conclusion, current treatment methods for neuroblastoma are generally ineffective and particularly toxic to these patients. Thus, there is a need for the development of effective therapeutic agents for these cancers with minimal toxicity. Our studies demonstrated that NM significantly inhibited the growth of xenograft tumors derived from the neuroblastoma cell line SK-N-MC *in vivo* and significantly inhibited cell proliferation and induced apoptosis *in vitro*. In addition, invasive parameters, such as MMP-2 and -9 secretion, in the SK-N-MC cell line were significantly inhibited by NM *in vitro*, while TIMP-2 was enhanced. These findings suggest the potential of NM for the treatment of neuroblastoma. Furthermore, in contrast to the toxic side effects of chemotherapy, the nutrient mixture was shown to be a safe therapeutic agent. In a previous *in vivo* study addressing safety issues, we found that gavaging adult female ODS rats (weighing 250–300 g) with the nutrient mixture (at 30, 90 or 150 mg/day for 7 days), had neither adverse effects on vital organs (heart, liver and kidney) nor on associated functional serum enzymes, indicating that this mixture is safe to use even at high doses, which far exceed the normal equivalent dosage of the nutrient (31).
